# Preexisting Mental Disorders and Mental Distress During the Pandemic: The Roles of Stress, Risk Perception, and Loneliness

**DOI:** 10.21203/rs.3.rs-4595482/v1

**Published:** 2024-07-16

**Authors:** Soyoung Kwon

**Affiliations:** The University of Texas at Dallas

**Keywords:** Mental disorders, comorbidity, adversities, pandemic

## Abstract

**Purpose.:**

The COVID-19 pandemic has imposed unprecedented stressors on individuals globally, leading to significant mental health challenges. This study examines the relationship between perceived adversities experienced during the pandemic—such as stress, risk perception, and loneliness—and the mental health outcomes of individuals with a specific focus on those already grappling with mental disorders.

**Methods.:**

The study analyzed data from 8,259 adults who participated in surveys between waves 4 (April 2020) and 29 (June 2021) of the Understanding American Study. Participants self-reported their psychiatric diagnosis history and completed self-report measures of mental distress (PHQ-4), perceived stress, risk perception, and loneliness.

**Results.:**

Those with preexisting comorbid mental disorders reported higher levels of mental distress, COVID-19 risk perception, perceived stress as well as more days experiencing loneliness during the pandemic. Perceived adversities—stress, risk perception, and loneliness—were all positively associated with increased mental distress, indicating the risk factors for mental distress during the pandemic. Among these factors, loneliness was identified as the primary contributor, accounting for 30%-40% of the mental health gap between individuals with and without preexisting mental diagnoses. Also, the detrimental impact of these adversities was more pronounced for those with preexisting mental disorders.

**Conclusion.:**

The research highlights that those with preexisting mental disorders, particularly those with comorbidities, face an increased risk of experiencing mental distress during the COVID-19 pandemic. These findings underscore the critical importance of providing targeted support and interventions specifically designed for this vulnerable population, with a particular focus on addressing loneliness.

## Introduction

Although the pandemic has caused widespread mental health problems [1, 2], it has disproportionately affected certain segments of the population, particularly those with preexisting mental disorders [3, 4]. A growing body of studies indicates significantly higher levels of mental distress across various types of mental health disorders, during the pandemic compared to the general population [3–10], suggesting that the presence of mental disorders itself acts as a risk factor for experiencing heightened mental distress during the COVID-19 pandemic. On the other hand, some longitudinal studies have reported minimal to no change in the symptomology in this population [11–13]. For instance, a large Dutch study found no significant difference in symptoms between psychiatric groups and controls during the pandemic [11, 12]. Similarly, a study in the U.S. revealed that individuals diagnosed with mental health disorders experienced a rise in distress levels during the pandemic, comparable in magnitude to that observed in the general population [7]. However, it is important to note that those with mental disorders exhibit significantly higher levels of depression, anxiety, loneliness, and COVID-related fear compared to healthy controls in absolute terms [14].

It is well established that experiencing stressful life events heightens the likelihood of relapse in adults who have already been diagnosed with mental disorders [15]. In the context of the pandemic, the widespread public health crisis and its disruptive impact on daily life represent significant stressors. Specifically, the emotional toll of the pandemic, coupled with measures like social distancing and quarantine enacted to control its spread, has significantly increased the vulnerability of individuals already grappling with mental disorders. This heightened vulnerability poses a greater risk for various adverse outcomes, including the onset of new symptoms, relapse of psychotic symptoms, or exacerbation of existing conditions [5, 8, 16–18]. Further, disruptions in access to mental health care and obstacles to receiving adequate social support during the pandemic further compound the risk of deteriorating mental health outcomes [19, 20]. Notably, individuals with mental disorders may be particularly susceptible to the adverse effects of social isolation, loneliness, stress, worries, and fears, all of which have been amplified by the COVID-19 pandemic [19, 21]. This increased susceptibility can lead to symptom relapse or deterioration among those already dealing with mental health challenges [6, 14, 19, 20].

Despite widespread concern that individuals with preexisting psychiatric conditions are particularly at risk during the pandemic [19, 20], a notable gap persists in understanding the differential impacts on these individuals compared to those without such conditions. It is crucial to identify the psychological impact of COVID-19 among psychiatric patients and to determine those most at risk, enabling the development of effective mitigation strategies for current and future outbreaks. This gap may stem from the prevailing assumption that individuals with preexisting mental disorders are inherently at a heightened risk for poor mental health outcomes. However, this assumption oversimplifies the complex interaction between preexisting mental disorders and the unique stressors posed by the pandemic. While pre-existing mental disorder was a risk factor for mental distress [22], the pandemic introduces a myriad of factors that can impact mental health outcomes in unpredictable ways. Therefore, delving deeper into the specific experiences of individuals with preexisting mental disorders during the pandemic is important to understanding how these factors intersect and influence mental health outcomes.

The present study examines the psychological well-being of U.S. adults during the COVID-19 pandemic, with a specific focus on individuals who have reported a preexisting mental health diagnosis. While extant research has primarily documented the mental health consequences of the pandemic for those with preexisting mental disorders, little is known about the mechanisms that place them at greater risk for mental distress during this time. This study specifically explores how adversities such as perceived stress, risk perception, and loneliness experienced during the pandemic act as mediators within the stress process framework and as amplifiers within the stress amplification framework, affecting the mental well-being of those already grappling with preexisting mental disorders. Drawing on data from 24 waves of the Understanding America Study spanning from April 2020 to June 2021, this study investigated how risk perception, stress, and loneliness intersect within this population. The specific research aims to: 1) Identify differences in mental distress and perceived adversities between individuals with and without preexisting mental disorders during the pandemic; 2) investigate whether perceived adversities as risk factors - namely perceived stress, risk perception, and loneliness – contribute to the mental distress experienced by those with preexisting mental disorders; and 3) assess whether preexisting mental disorders the adverse effects of these perceived adversities on mental health.

### Perceived adversities and mental health during the pandemic: Stress, risk perception, and loneliness

Risk perception, an individual’s individual assessment of the risk associated with a specific hazard (e.g., health threat), has been consistently linked to heightened psychological distress and negative emotional states [23–25]. While risk perception during the pandemic has primarily focused on the general population in the extant literature, some studies highlighted the prevalence of intensified risk perception among psychiatric patients [10, 17]. However, there is evidence pointing to a bidirectional relationship between mental health and risk perception, suggesting that existing mental disorders could shape an individual’s perception of risk, particularly in the context of the COVID-19 pandemic [26]. Whether this relationship goes in either direction, it becomes imperative to consider risk perception in understanding mental health in individuals with preexisting mental disorders during the pandemic.

Perceived risk often triggers feelings of stress, but the perception of stress can vary greatly among individuals even when confronted with large-scale societal stressors such as a pandemic, war, or economic recession that affects the entire society. These objectively stressful events often have inequitable effects on different segments of society, as their impacts largely hinge on an individual’s perception of the stress. This perceived stress is influenced by factors such as the individual’s available resources (e.g., financial stability, social support, access to health care, etc.) and their sociodemographic characteristics [27]. For example, people with mental health disorders like anxiety and depression experience greater stress during the pandemic [4, 6, 21].

Studies have consistently documented that loneliness resulting from social isolation increases the risk of experiencing higher levels of anxiety and depression [28, 29]. Moreover, loneliness had already reached epidemic proportions even before the pandemic [30]. Thus, there has been growing public health concern that the pandemic has exacerbated this issue, as loneliness has emerged as a significant risk factor for mental health due to the intensified social distancing and quarantine measures during the pandemic [18, 31, 32]. Research indicated that loneliness is more prevalent among individuals with mental health issues compared to the general population both before and during the pandemic [18, 33–35]. The determinantal effect of loneliness on mental health, well documented in the literature, may be especially pronounced for individuals with preexisting mental illnesses who often grapple with loneliness and social isolation [32]. Indeed, Goh et al. [31], in their study conducted in Japan during the pandemic, found that patients with mental disorders were more vulnerable to moderate to severe loneliness and high social isolation, exacerbating their existing mental illness symptoms.

### Theoretical Perspectives

The present study grounds the investigation within the theoretical frameworks of stress process and stress amplification. The stress process theory offers a lens through which we understand stress as a dynamic unfolding process influenced by various factors like environment, stressors, resources, and outcomes [36]. Specifically, individuals with preexisting mental disorders may perceive the threat of COVID-19 as more immediate and severe due to their existing vulnerabilities. This heightened perception of adversities can trigger stress, compounding their existing mental health struggles. It is anticipated that stress, risk perception, and loneliness—emerging as significant secondary stressors—mediate the relationship between preexisting mental disorders and elevated mental distress. Moreover, the stress amplification theory sheds light on how existing vulnerabilities can amplify the impact of stressors, leading to more severe outcomes. Stress amplification, defined as the experience of exacerbated strain due to multiple stressors, underscores the compounding effect of stressors on mental health [37]. For individuals with mental disorders, the COVID-19 pandemic serves as a significant stressor that can exacerbate their mental health distress. Essentially, preexisting mental disorders may act as predisposing risk factors, intensifying vulnerability to the impact of additional stressors. In such a scenario, the phenomenon of stress amplification comes into play, magnifying the effects of stress on their mental well-being.

## Methods

### Data and Sample

This paper draws upon data from the Understanding America Study (UAS) COVID-19 Survey, a nationally representative probability sample of non-institutionalized U.S. adults ages 18 and older (Holmes et al., 2020). Participants were recruited randomly through address-based sampling with the US Postal Service Computerized Delivery Sequence (CDS) file households that cover almost all of U.S. households [38]. As the surveys were conducted online and internet access was necessary, participants who may otherwise not be able to participate in the survey were provided internet access and a tablet [38]. Approximately 7,000 out of the 9,000 UAS panel members consented to participate in the UAS COVID-19 Tracking Survey [39].

The current study utilized de-identified, publicly available data from the UAS and was deemed exempt from institutional review board ethics approval. While the first wave was collected from March 10 through March 31, the present study focused on data spanning 26 waves from April 2020 (Wave 4) to June 2021 (Wave 29), comprising a total of 157,117 observations from 8,259 unique participants who contributed to at least one of these waves. Approximately 50% of participants were observed in 24 waves or fewer, while 25% participated in all 26 waves. The exclusion of the first three waves was necessary due to the lack of key questions related to pre-existing mental disorders, perceived stress and loneliness.

### Measures

#### Pre-existing mental disorders and comorbidity.

The presence of preexisting mental disorders was assessed using a response to the question of whether health professionals had diagnosed them with specific disorders such as anxiety, attention-deficit hyperactivity disorder (ADHD), bipolar disorder, eating disorder, depressive disorders, obsessive-compulsive disorder (OCD), post-traumatic stress disorder (PTSD), or schizophrenia/psychotic disorder, or any other mental disorders. The responses include yes, no, or unsure. Those who answered “yes” were further queried about the timing of the diagnosis, specifically if it occurred before March 10, 2020.

Extant research has often focused on specific types of preexisting mental disorders or the presence of any preexisting mental disorder. However, relatively few studies have explicitly considered the aspects of comorbidity, with some exceptions. Some studies have shown a positive association between comorbidity and increased mental distress during the pandemic compared to individuals with a single mental disorder [12, 40]. In response to this, this study established a variable termed “burden of preexisting mental disorders,” which indicates the count of mental diagnoses respondents had ever diagnosed. The variable for preexisting mental disorders was categorized into three groups: no preexisting mental disorder, one preexisting mental disorder, and two or more preexisting mental disorders (i.e., comorbidity of preexisting mental disorders).

#### Mental distress.

Respondents were asked a 4-item Patient Health Questionnaire (PHQ-4) for mental health, specifically anxiety and depression, and validated for use in both clinical and non-clinical samples [41, 42]. Specifically, the PHQ-4 comprises the following four symptoms over the past 14 days: (1) “ Over the past fourteen days, how often have you been bothered by any of the following problems? (1) Feeling nervous, anxious, or on edge; (2) not being able to stop or control worrying; (3) feeling down, depressed, or hopeless; and (4) having little interest or pleasure in doing things.” Items were scored on a scale of 1 “not at all” to 4 “nearly every day” (α = 0.88 in the current sample). The total score ranged from 0 to 12, with higher scores indicating greater mental distress.

#### Perceived adversities.

Perceived stress, loneliness, and perceived risk were used to indicate perceived adversities during the pandemic [43]. Perceived stress was assessed using four items from the Perceived Stress scale (PSS-4) [44]. Participants were asked how often in the past fourteen days they felt: (1) unable to control the important things in your life; (2) confident about your ability to handle personal problems; (3) that things were going your way; and (4) difficulties were piling up so high that you could not overcome them. Responses were rated on a scale from 0 (*never*), 1 (*almost never*), 2 (*sometimes*), 3 (*fairly often*), to 4 (*very often*). The second and the third questions were reversed scored. The total score, ranging from 0 to 16, indicated higher levels of perceived stress with higher scores.

The perceived risk of COVID-19 was measured. Based on the perceived risk of death from COVID-19, contracting COVID-19, job loss due to COVID-19, and financial strain in the next three months. Each item was rated on a scale from 0% to 100%. The overall perceived risk score was calculated by averaging these items and then dividing by 10, resulting in a score range from 0 to 10.

Loneliness was assessed with a single question: “In the past 7 days, how often have you felt lonely?” Response options were 1 (*not at all or less than 1 day*), 2 (*1–2 days*), 3 (*3–4 days*), and 4 (*5–7 days*).

#### Covariates.

Covariates included age, gender, educational level, race/ethnicity, marital status, household income, a number of chronic conditions, working status, U.S citizenship status, nativity, and immigrant status along with the survey wave.

### Statistical analyses

First, I analyzed the sample characteristics for the entire sample, as well as two subsets, individuals with and without preexisting mental disorders. Next, I conducted a series of mixed effects models that accommodate missing responses and the correlated nature of repeated measurements. These models were examined separately for each key variable – mental distress, perceived stress, risk perception, and loneliness- resulting in four distinct models. In the second part of the analysis, I estimated the effect of perceived adversities on mental distress. Model 1 included the comorbidity of preexisting mental disorders as an independent variable while controlling for all covariates. Model 2 added measurements of perceived adversities. Finally, Model 3 included two-way interactions between perceived adversities and the comorbidity of preexisting mental disorders. All analyses incorporated survey weights and used Stata 18.0 [45].

## Results

Table 1 presents the descriptive statistics of the variables, calculated at the person-wave level for those with and without preexisting mental disorders, as well as for the overall sample. About 29% of the participants reported being diagnosed with at least one mental disorder before March 2020, with 15% reporting comorbid mental disorders. During the pandemic, 5%-6% of the respondents reported experiencing clinical levels of depression and anxiety, while over 20 % of respondents with mental disorder diagnoses experienced these clinical levels. Those with at least one diagnosed mental disorder also reported higher levels of mental distress, stress, risk perception, and loneliness.

**Figure 1:**
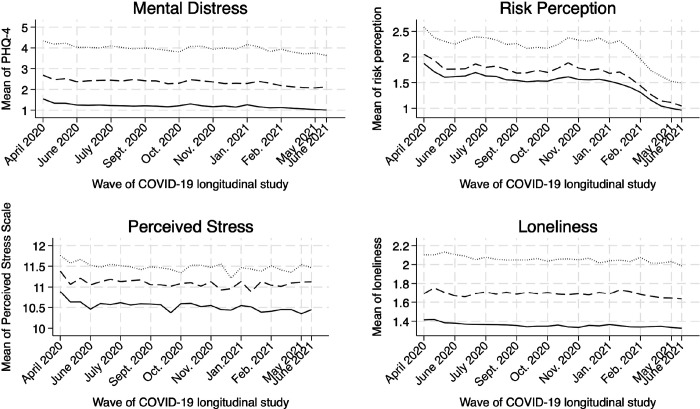
Unadjusted means of mental health and perceived adversities over time by pre-existing mental health diagnosis status, Understanding America Study (UAS). April 2020-June 2021

As shown in Fig. 1, the differences in these variables across comorbid mental disorders remained consistent over time: individuals with more mental health disorders reported greater mental distress, higher perceived stress, increased risk perception, and more days of loneliness throughout the pandemic.

### Gradients in mental distress and perceived adversities by preexisting mental disorders

**Figure 2:**
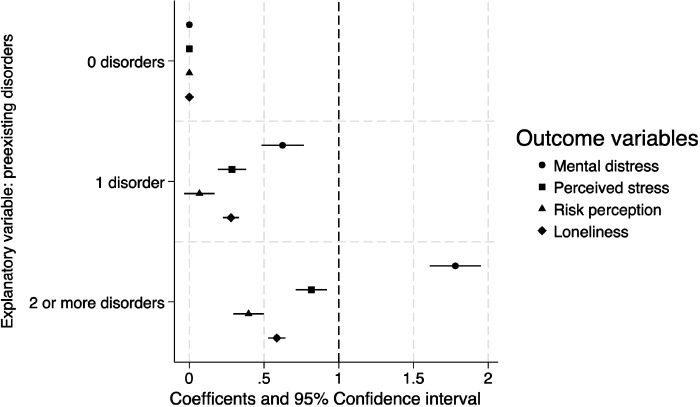
Multivariable mixed effects regression models predicting mental distress and perceived adversities based on pre-existing mental health diagnosis status. Note: Figure 2 is based on the mixed effects regression models that evaluate the adjusted associations of pre-existing mental health diagnosis status with mental distress and perceived adversities, controlling for the covariates. The vertical line represents the null coefficient (coefficient = 0), while the bars denote the 95% Confidence Intervals. Each panel represents a single model predicting mental distress, perceived stress, risk perception, and loneliness, respectively.

Mixed effects regression results suggest that having preexisting comorbid mental health disorders is positively associated with increased psychological health problems. The variable representing the comorbidity of the mental disorders exhibited a positive graded response relationship. Individuals with multiple preexisting mental disorders reported higher levels of mental distress, greater COVID-19 risk perception, increased perceived stress, and more days of experiencing loneliness during the pandemic (Fig. 2).

### Role of perceived adversities in the association between preexisting mental disorders and mental distress

Moving to mixed effects models predicting mental distress, the results are presented in Table 2. Covariates are suppressed for sake of space, but the full set of estimates is available in Supplementary Material. In Model 1 of Table 2, individuals with preexisting mental disorders exhibited higher levels of mental distress compared to those without any disorder, which was already displayed in panel A of Figure 2 above. Model 2 of Table 2 expands upon this by incorporating measures of perceived adversities – perceived stress, COVID-19 risk perception, and loneliness. With these additional variables of perceived adversities, the coefficients of pre-existing mental disorders decrease. For instance, the coefficient for two or more comorbidities in preexisting mental disorders decreases from 1.786 to 1.206. This indicates that perceived adversities account for approximately 30 % of the mental health disparity between individuals with comorbidity of mental disorders and those without such disorders and approximately 40% of the mental health gap between those with one preexisting mental disorder and those without such disorders. Additionally, each variable of perceived adversity was positively associated with increased mental distress. Using the ML mediation approach by Krull and MacKinnon [46], I examined which adversities variables mediate the association, revealing that loneliness primarily accounts for 27%-35% of the mental health gap between those with and without mental disorders.

**Figure 3:**
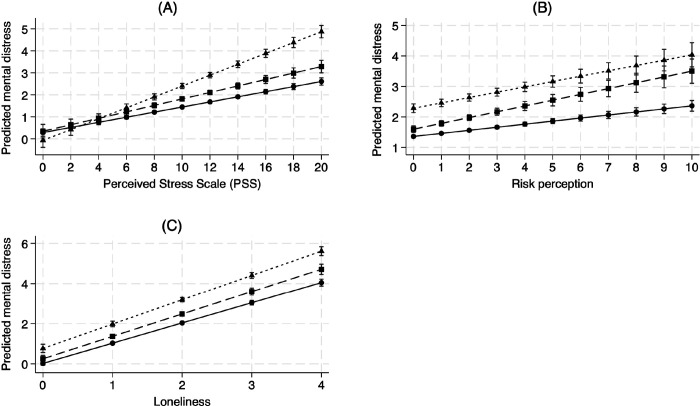
Mental distress and perceived adversities by pre-existing mental health diagnosis status Note: (A) Predicted mental distress by stress and preexisting disorders; (B) Predicted mental distress by risk perception and preexisting disorders; (C) Predicted mental distress by loneliness and preexisting disorders; Figure 3 is based on Model 3 of Table 2.

The addition of interaction terms between comorbid mental health conditions and perceived adversities in Model 3 showed that people with preexisting mental disorders experience greater mental distress than those without disorders when faced with greater levels of stress, risk perception, and loneliness. Those with comorbid mental health disorders had steeper slopes for stress, perceived risk, and loneliness, indicating that their distress levels were more significantly impacted by these perceived adversities (See Fig. 3). The effects of perceived adversities were most detrimental for those with comorbid mental disorders.

## Discussion

It has been widely suggested that COVID-19 could profoundly impact individuals with preexisting mental disorders [19, 20]. However, research on this topic has been limited. This study addresses this gap by examining differences in mental distress and perceived adversities between those with and without preexisting mental disorders during the pandemic.

The findings indicate that the COVID-19 pandemic has disproportionately affected people with preexisting mental disorders in terms of stress, risk perception, and loneliness, as well as mental distress. The finding echoes previous studies showing that psychiatric conditions present before the pandemic are associated with worse anxiety and depression symptoms [3, 6, 8, 12, 18]. Our ancillary analysis found no statistical significance in the interaction terms between survey waves and preexisting mental disorders, indicating that the changes in mental distress over time during the pandemic were similar for both groups. Similarly, Pan et al. [12] reported that people with preexisting mental disorders in the Netherlands exhibited higher mental distress, fear, and poor coping both before and during the pandemic, but did not show a greater increase in symptoms. This suggests that individuals with preexisting mental disorders consistently faced heightened mental challenges throughout the pandemic compared to those without such disorders.

It is worth noting that without a pre-pandemic baseline, the present study cannot accurately gauge how mental health shifted for different groups during the outbreak. Previous studies offer mixed findings. For instance, Kwong et al. [47] found that anxiety and depression remained high in those with existing mental disorders, even after adjusting for initial symptoms, In contrast, another study observed improvement in individuals with pre-existing conditions, while those without preexisting mental health challenges experienced heightened psychological distress in the context of the pandemic [48]. Similarly, Pan et al. [12] noted a slight reduction in symptoms among those with the highest mental health burdens pre-pandemic. These varied findings hint at the complex interplay between prior mental health conditions and pandemic-induced psychological effects. Future studies are needed to compare changes in mental health before and during the pandemic further to understand these dynamics better. Such studies could elucidate the trajectory of mental health symptoms across different populations, guiding targeted interventions.

This study also highlighted that the comorbidity of preexisting mental disorders was significantly associated with mental distress symptoms, further emphasizing the cumulative impact of multiple mental health conditions. Ancillary analysis with six categories of preexisting mental disorders (0, 1,2,3,4, and 5 or more comorbidity in mental disorders) consistently indicated a positive dose-response [12, 49]. This pattern underscores the importance of addressing multiple mental health conditions simultaneously, as the combined burden can significantly exacerbate mental health issues during crises like the COVID-19 pandemic.

Another key finding is that perceived adversities partially account for the association between preexisting mental disorders and mental distress. Individuals with preexisting mental disorders may experience heightened perceived stress due to their baseline vulnerability. Similarly, a high-risk perception of COVID-19 can increase perceived stress, particularly among those with mental health disorders, who are already predisposed to anxiety and fear [50]. Notably, loneliness emerged as a critical contributor to mental distress in individuals with preexisting mental disorders. The enforced isolation and social distancing measures during the pandemic likely intensified feelings of loneliness, which in turn exacerbated their mental distress [18, 32, 34], and individuals with preexisting mental disorders might feel this isolation more acutely due to their reliance on social interactions for support [31, 32].

Perceived adversities are recognized as risk factors development of depression and anxiety [43] and as psychological responses to the challenges posed by the pandemic. High-risk perception, or the fear of contracting COVID-19, may cause hypervigilance and worry, contributing to anxiety and worsening depression. Similarly, loneliness, a known risk factor for depression, can lead to feelings of worthlessness and sadness, potentially spiraling into clinical depression. The perceived adversities also reflect how individuals mentally and emotionally process external stressors like health risks, economic instability during the pandemic, social distancing measures, and information about the pandemic. For example, social isolation measures, such as lockdowns and physical distancing, have increased feelings of loneliness.

The interactional model suggested that the impact of perceived adversities was intensified for those with preexisting mental disorders, showing a stress amplification pattern. Their existing mental health challenges may make them less equipped to manage additional stress, leading to greater mental distress. In essence, individuals with preexisting mental disorders face a compounded burden: their baseline vulnerability to stress is exacerbated by the intensified perception of adversities during the pandemic. This underscores the need for targeted interventions that address the heightened sensitivity and unique challenges faced by this population, aiming to mitigate the amplified effects of stressors and support their mental well-being during such crises.

Interestingly, individuals with preexisting mental disorders exhibit lower mental distress when there’s no perceived stress, but experience a stronger impact when stress is present, plausibly aligning with the Stress Susceptibility Model. This model suggests they have a lower stress tolerance threshold, making them more vulnerable to stressors [51]. In the absence of stress, their coping mechanisms could maintain a stable, potentially lower level of distress. However, when stress arises, their susceptibility leads to a disproportionate increase in distress as their coping resources are overwhelmed. Ancillary analysis reveals they tend to use relaxing (e.g., exercise, meditation) and maladaptive strategies (e.g., cannabis, alcohol), supporting the idea that their coping mechanisms contribute significantly to their heightened response to stress. Further research is needed to explore coping mechanisms in this population during the pandemic.

Despite this study’s contributions to the literature concerning mental health, psychiatric disorders, and the pandemic, several limitations should be acknowledged. Firstly, the use of self-reported measures may introduce response bias, particularly regarding mental distress and the history of mental disorder diagnoses, which could be underreported due to social desirability bias. Future research could benefit from incorporating structured interviews and evaluations by others, such as teachers and friends, to assess the psychological state of individuals more accurately. Another limitation is the inability to disentangle the extent to which anxiety and depression symptoms are directly due to the pandemic or were preexisting conditions exacerbated by it, given the absence of pre-pandemic data. Considering the elevated rates of anxiety and depression symptoms that prevailed before the pandemic, especially in those with diagnosed mental disorders, having baseline estimates would have been important to fully understand the effects of the pandemic. Nonetheless, the present study highlights the increased vulnerability of individuals with preexisting mental disorders, which can be attributed to their heightened susceptibility to stress, risk perception, and loneliness. These findings underscore the importance of addressing the unique needs of this population, particularly during times of public health crises. Finally, though the present study explicitly considered comorbidity with a number of disorders, it did not take into account the chronicity of these disorders. Beyond comorbidity, chronicity could be a significant factor in understanding the mental health impacts on psychiatric patients during the pandemic as long-term sufferers may have developed different coping mechanisms or may experience more severe symptoms compared to those with more recent diagnoses.

In conclusion, the sample of respondents who reported preexisting mental health diagnoses were more vulnerable to mental distress, stress, loneliness, and risk perception related to COVID-19. Addressing the mental health needs of those with preexisting conditions is critical in reducing the overall burden of mental distress during public health emergencies. Future research should focus on the long-term effects of such crises on mental health and develop effective strategies to support those most at risk.

## Figures and Tables

**Table 1.** Descriptive statistics of the variables included in the analysis, calculated at the person-wave level, UAS (April 2020– June 2021)

	Overall (N=8341)		No diagnosis (N=5776)		Diagnosed (N=2553)	
Variable			Mean/%	Std. Dev	Mean/%	Std. Dev
Preexisting mental disorders						
1 mental disorders (no diagnosis)	71.09%					
1 mental disorder	13.53%				47%	
2 or more mental disorders	15.38%				53%	
Mental distress (PHQ-4, 0–12)	1.79	2.53	1.20	1.98	3.22	3.02
Depression (PHQ-4 ≥ 3)	10.35%		5.77%		21.37%	
Anxiety (PHQ-4 Subscale, PHQ-4 ≥ 3)	11.18%		6.61%		22.19%	
Perceived stress scale (PSS, 0–20)	10.75	1.92	10.53	3.36	11.30	1.52
Loneliness (1–4)	1.51	0.78	1.36	0.64	1.88	0.93
Risk perception of COVID-19 (1–10)	1.63	1.58	1.50	1.54	1.94	1.68
No. chronic conditions (0–8)	1.01	1.21	0.88	1.11	1.33	1.35
Female (0=female, 1=male)	41.27%		45.98%		29.71%	
Age (in-years 18110)	51.55	16.34	52.35	16.69	49.60	15.33
Race/ethnicity						
White	67%		64%		73%	
Black	8%		9%		6%	
Hispanic	12%		13%		9%	
Asian	5%		7%		3%	
Pacific Islander or native American	1%		7%		1%	
Mixed	2%				2%	
Household income (level, 1–16)	11.35	4.100	11.65	4.0771	10.63	4.23
Marital status (married)	55%		59%		46%	
U.S citizen	97%		96%		99%	
U.S born	89%		87%		93%	
Education (level, 116)	11.39	2.27	11.4476	2.28969	11.26	2.24
Currently working	56%		58%		51%	
Immigrant status	46%		48%		42%	

**Table 2.** Mental distress regressed on preexisting mental disorders and perceived adversities, UAS (April2020– June 2021) Abridged (N=8, 259)

	Model 1	Model 2	Model 3
VARIABLES	Coef.	Coef.	Coef.
Preexisting mental disorder			
(Ref. No preexisting mental disorder)			
One mental disorder	0.624***	0.379***	−0.26
	(0.072)	(0.053)	(0.203)
Two or more mental disorders	1.780***	1.206***	−0.797***
	(0.087)	(0.066)	(0.205)
Perceived adversities			
Risk perception		0.129***	0.100***
		(0.009)	(0.01)
Perceived stress scale (PSS)		0.138***	0.116***
		(0.007)	(0.008)
Loneliness		1.087***	1.006***
		(0.024)	(0.031)
Interaction terms			
One disorder X Risk perception			0.090***
(0.025)			
Two or more disorders X Risk perception			0.075**
			(0.026)
One disorder X Perceived stress scale			0.031+
			(0.017)
Two or more disorders X Perceived stress scale			0.131***
			(0.016)
One disorder X Loneliness			0.108+
			(0.058)
Two or more disorders X Loneliness			0.206***
			(0.056)
Constant	6.371***	1.462***	1.826***
	(0.246)	(0.210)	(0.214)
Random-effects parameters			
Level 1 (occasions): residuals	2.194***	1.851***	1.840***
Level 2(individuals): Intercept	3.563***	1.949***	1.891***

*Note*: Robust standard errors in parentheses. Models include control for sociodemographic characteristics, chronic health conditions, and immigration and citizenship.

*** p<0.001

** p<0.01

* p<0.05

+ p<0.1

## Data Availability

The UAS data can be accessed via https://uasdata.usc.edu/index.php with UAS’s data user agreement.
